# Multiple Keratoacanthoma-like Syndromes: Case Report and Literature Review

**DOI:** 10.3390/medicina60030371

**Published:** 2024-02-22

**Authors:** Emmanouil Karampinis, Christina Kostopoulou, Olga Toli, Leonidas Marinos, George Papadimitriou, Angeliki Victoria Roussaki Schulze, Efterpi Zafiriou

**Affiliations:** 1 Department of Dermatology, University of Thessaly, University Hospital of Larissa, 41334 Larissa, Greece; ekarampinis@uth.gr (E.K.); roussaki@otenet.gr (A.V.R.S.); 2 Department of Dermatology, Trikala Hospital, 42131 Trikala, Greece; 3 Department of Dermatology, Oncoderm Center One Day Clinic, 45332 Ioannina, Greece; olgatolimail@gmail.com; 4 Department of Hematopathology, Evangelismos Hospital, 11527 Athens, Greece; lestrand@yahoo.gr; 5 Department of Plastic Surgery, IASO Hospital, 41005 Larissa, Greece; gppdm@yahooo.gr

**Keywords:** keratoacanthoma, Ferguson–Smith syndrome, Muir–Torre syndrome, eruptive keratoacanthoma of Grzybowski, multiple familial keratoacanthoma of Witten and Zak, incontinentia pigmenti

## Abstract

Keratoacanthoma (KA) is a fast-growing skin tumor subtype that can be observed as a solitary lesion or rarely as multiple lesions in the context of rare genetic syndromes. Syndromes with multiple keratoacanthoma-like lesions have been documented as multiple self-healing squamous epithelioma (Ferguson–Smith syndrome), eruptive keratoacanthoma of Grzybowski, multiple familial keratoacanthoma of Witten and Zak Muir–Torre syndrome, and incontinentia pigmenti. The treatment approach of those entities is challenging due to the numerous lesions, the lesions’ undefined nature, and the co-existence of other malignant skin tumors. Herein, we report a case of a 40-year-old woman who developed multiple treatment-resistant Ferguson–Smith-like keratoacanthomas with a co-existing large and ulcerated invasive squamous cell carcinoma and microcystic adnexal carcinoma on the scalp. Multiple keratoacanthomas on her extremities were successfully treated with oral acitretin (0.5 mg/kg/day) in combination with topical Fluorouracil (5-FU) 5%, while excision and plastic surgery restoration were performed to treat the ulcerated cancer lesion on her scalp. Due to the interesting nature of this rare syndrome, we performed a literature review including case reports and case series on multiple-KA-like lesions syndromes and focusing on diagnosis and therapy approaches. We also conducted a comparison of patient reports, which included assessing the clinical appearance of the lesions and evaluating the success and progress or the failure of various treatment approaches that were implemented.

## 1. Introduction

Keratoacanthoma (KA) is a fast-growing skin tumor subtype that typically develops from cells within hair follicles on sun-exposed skin. In most cases, the lesions last approximately 4 to 6 months until regression, and they include three distinct phases, namely, the proliferative stage, the mature stage, and the involutional stage. Their macroscopic appearance includes a sharply defined, firm lesion that can appear either reddish or similar in color to the surrounding skin. They typically feature a distinctive thickened plug at the center with a smooth perimeter. Removing this keratinized central core results in a depression or “crater” appearance [1]. Its appearance may differ in ethnic skin [2]. The clinicians usually focus on the comparison between keratoacanthoma and squamous cell carcinoma (SCC), with the second one being a more aggressive subtype of skin cancer with a greater tendency for metastases. Keratoacanthoma is considered to be an SCC subtype, although there is still debate on the nature of the lesion [3]. Dermoscopy can assist in the clinical differentiation of KA from other lesions, yet it may not consistently differentiate KA from squamous cell carcinoma. The central keratin plug is a dermoscopic characteristic with discriminative ability [1]. Concerning genetic differences between KA and SCC, most studies found no differences between the two lesions. However, microenvironment genetics showed differences mostly in genes controlling neovascularization, while different gene expressions were observed depending on neoplasia size mainly on SCC [1].

Keratoacanthomas can appear as solid lesions or multiple lesions in the case of rare genetic syndromes. In the first group mentioned, apart from the solitary keratoacanthoma, which is the most common form, there is the giant keratoacanthoma (usually > 2 cm) and the keratoacanthoma centrifugum marginatum. A keratoacanthoma centrifugum marginatum can be distinguished by its outward growth around the edges and the central healing process accompanied by scarring, often without a tendency to naturally resolve [4]. Genetic syndromes that multiple keratoacanthoma-like lesions can occur in are Muir–Torre syndrome, multiple self-healing squamous epithelioma (Ferguson–Smith syndrome), eruptive keratoacanthoma of Grzybowski, multiple familial keratoacanthoma of Witten and Zak, and incontinentia pigmenti. 

Muir–Torre syndrome represents an autosomal dominant manifestation of hereditary non-polyposis colorectal cancer, commonly known as Lynch syndrome. It is characterized by the development of sebaceous skin tumors and various internal malignancies, with colon cancer being the most prevalent. In this syndrome, sebaceous characteristics can even be discerned within a keratoacanthoma [5]. Multiple self-healing squamous epithelioma (MSSE) of Ferguson–Smith are rare, autosomal, dominantly inherited diseases with recurrent, histologically malignant tumors that undergo spontaneous regression. Those lesions resemble KA. However, multiple self-healing epitheliomas can usually be distinguished from keratoacanthomas because of the multiple lesions, the earlier ages of the disease onset, and the familial nature of the condition, but a definite diagnosis, however, as in almost all genetic disorders, can be made with genetic testing (such as Transforming Growth Factor Beta Receptor 1 (TGFBR1) mutations). Histologically, compared with keratoacanthoma, there is no marked “shouldering”, and the leucocyte abscesses are absent. Multiple self-healing epitheliomas, before being recognized as a disease entity, were sometimes mistaken for malignant squamous carcinomas treated with wide surgical excision and radiotherapy, leading, in the first case, to death and, in the second, to aggressive cancer transformations [6]. 

In the case of eruptive keratoacanthoma of Grzybowski, the syndrome is characterized by commencement in adulthood (typically during the fifth to seventh decade of life); a widespread outbreak of numerous; clearly outlined itchy papules, some of which have a hardened center; and a continuous and worsening course. The histopathological findings align with keratoacanthoma. Additionally, the presence of mucosal lesions and ectropion (outward turning of the eyelids) have been suggested as added indicators [7]. Multiple familial keratoacanthoma of Witten and Zak is a rare, autosomal, dominantly inherited variant of KA that presents in childhood with overlapping features of the Ferguson–Smith and Grzybowski subtypes [8]. Finally, patients with incontinentia pigmenti can present with skin symptoms that can be delineated into three successive phases: an initial stage characterized by blistering and the formation of vesicles and bullae, followed by a stage marked by wart-like growths, and finally, a phase exhibiting a swirling pattern of pigmentation. This category of patients presents with keratoacanthoma developing mainly on a pigmented patch [9].

Due to the heterogeneity of those syndromes and the nature of the syndrome-associated lesions (Table 1), different treatment strategies were followed in each case, including systemic therapy, topical applications, as well as surgical procedures. Therefore, we present a case of multiple cutaneous epitheliomas and its therapy approach. Following, we present case reports of keratoacanthoma-like syndromes with a special focus on the treatment strategies.

## 2. Case Report

**Figure 1:**
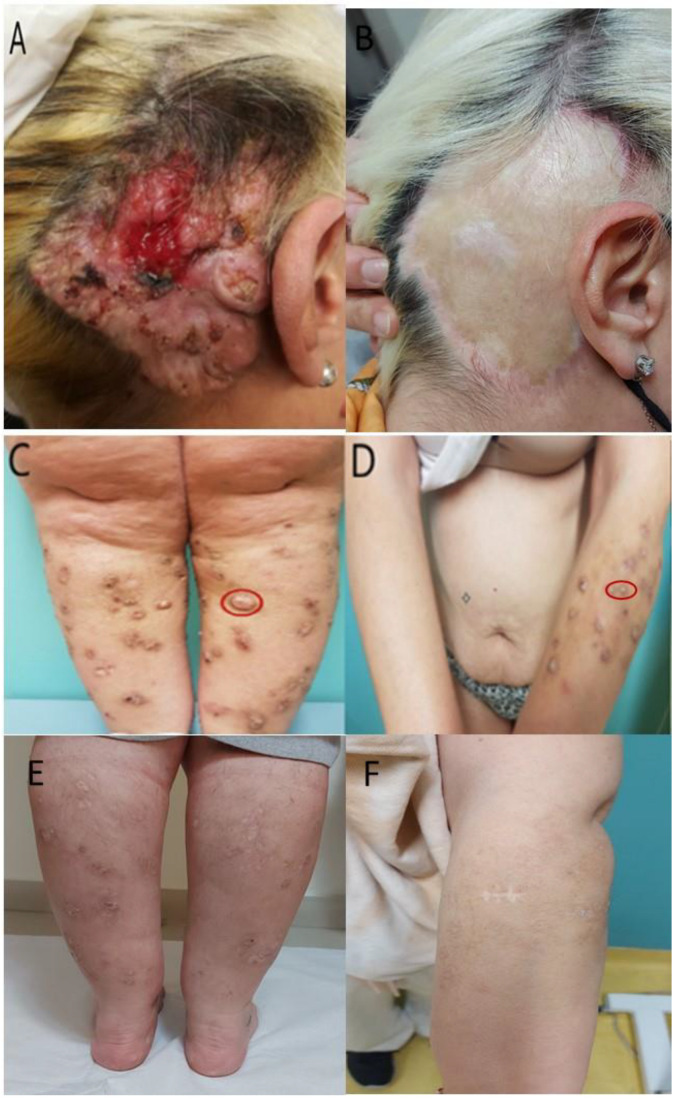
(**A**) Image showing an extensive ulcer with a nearby nodule expanding in the retroarticular area covering a large portion of the head skin. (**B**) Image showing the plastic surgery restoration after the removal of the lesion area. (**C**,**D**) Images showing dome-shaped, circumscribed, hyperkeratotic nodules with a skin-colored periphery located mainly on the front surface of the knees and arms. The lesion in the circle was biopsied. (**E**,**F**) Image showing the regression of the lesions after treatment with cryotherapy in combination with topical treatment with cream 5 FU 5% as well as acitretin (0.5 mg/kg/day).

Herein, we present a 40-year-old woman with Fitzpatrick skin type III. She visited the dermatology department due to the appearance of an extensive ulcerated lesion in the retroarticular area with expansion to the head. The lesion has changed shape and size over the years. The patient also presented with dome-shaped, circumscribed nodules with a skin-colored periphery and malodorous secretion. These lesions were accompanied by intense pruritus and were located mainly on the front surfaces of the knees and arms (Figure 1). The patient reported that the development of similar pruritic nodules on the upper and lower extremities occurred 20 years ago, but spontaneous healing of the lesions and scar formation followed. Concerning the ulcerated lesion, our patient mentioned a scarring post-traumatic injury in infancy and the appearance of exophytic lesions 10 years ago in the affected area, but in the last 2 years, the lesion tended to increase in periphery and resulted in central ulceration.

**Figure 2:**
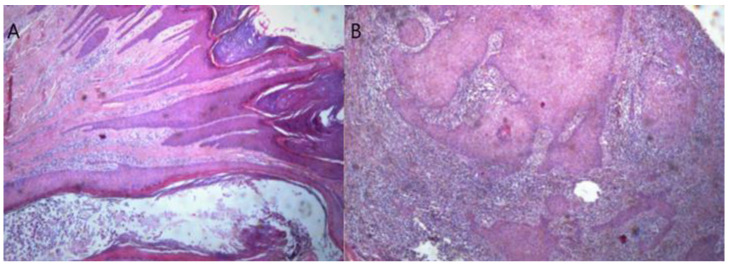
(**A**) Epidermal hyperplasia with irregular acanthosis, hyperkeratosis, follicular plugging, and multiple horny cysts (H&E × 20) (**B**) Deeper sections with minimally invasive areas (greater magnification, H&E × 40).

The patient family history revealed that her father had similar lesions on the arms and legs. Physical examination revealed the presence of multiple lesions on her left arm and bilaterally on her lower legs and body. The lesions ranged in size from 5 mm to 2.5 cm. Numerous lesions were present as hyperkeratotic nodules or plaques characterized as well demarcated, verrucous, and exophytic. Some lesions were very itchy, without regression observed, with atrophic scars on her arms and legs revealing older lesions. Two lesions were excised, one from a hyperkeratotic nodule on her left arm which revealed a lichenoid inflammatory reaction and the other obtained from an exophytic, verrucous nodule on her left anterior tibial surface (shin). Histology revealed epidermal hyperplasia with irregular acanthosis, hyperkeratosis and follicular plugging with areas resembling small keratoacanthomas and others more closely resembling a histopathology of well-differentiated squamous cell carcinoma. In the deeper sections, the lesion has an infiltrative, minimally invasive character with an intense inflammatory reaction (minimally invasive squamous cell carcinoma) (Figure 2).

As for the ulcerated lesion, a total surgical excision was performed, and the histopathological examination of the sample revealed a well-differentiated invasive squamous cell carcinoma combined with a microcystic adnexal carcinoma arising within a nevus sebaceous. Due to the clinical image of our patient and the histopathology findings of the skin lesions, a syndrome with multiple keratoacanthoma-like lesions was suspected, especially Ferguson–Smith (multiple self-healing squamous epitheliomas) or eruptive keratoacanthoma of Grzybowski due to the existence of itchy lesions.

The patient’s genetic investigation with whole-exome sequencing (WES) was per-formed to assess the following genes that are involved in squamous tumor development: COL17A1 (collagen type XVII alpha 1 chain gene), CYLD (gene which encodes a tumor suppressor of deubiquitinates called cylindromatosis with a predominant role in the regulation of Nuclear Factor-κB (NF-κB)), DICER1 (dicer 1, ribonuclease III gene), EWSR1 (gene whose product is an RNA-binding protein), KEAP1 (gene that encodes the kelch-like ECH-associated protein 1), TGFBI, TGFBR1, and TGFBR2. The investigation revealed no mutations in the above-mentioned genes.

At first, the patient was treated with topical ointment of salicylic acid 5% in Vaseline base, ointment of clobetasol 0.05%, and intralesional cortisone to the hyperkeratotic lesions without significant improvement. Finally, she was successfully treated with cryotherapy in combination with topical treatment with cream 5 FU 5%. Acitretin (0.5 mg/kg/day) was added, combined with the surgical removal of the larger lesions. The skin lesions on the extremities regressed without surgery 4 to 6 months after the initiation of acitretin, while further new lesions have not appeared. After 9 months of therapy, the patient presented with headache, nausea, and dry skin, and acitretin treatment was stopped. Regarding our patient’s follow up, she reported no new lesions in a period of one year after the acitretin discontinuation. As for the ulcerative lesion, after surgical removal, the patient underwent plastic surgery restoration (Figure 1).

In this challenging case, acitretin combined with topical chemotherapeutic agents such as 5-FU 5% or the surgical removal of larger lesions seems to be an effective treatment choice for a patient with keratoacanthoma-like lesions. The treatment scheme should be made taking into consideration the dose-dependent side effects of acitretin.

## 3. Methods

We performed a literature review dated from 2000 to 2023 searching for English-language-based cases of multiple keratoacanthoma syndromes and investigated each case’s diagnostic and therapeutic approach. The cases included were found by searching PubMed database using the following terms: [Muir–Torre syndrome] OR [Ferguson-Smith syndrome] OR [eruptive keratoacanthoma of Grzybowski] OR [multiple familial keratoacanthoma of Witten and Zak] OR [incontinentia pigmenti] AND [keratoacanthoma] AND [treatment]. Based on this search, we reported the accessible cases that involved patients with keratoacanthoma and or keratoacanthoma-like SCC as clinical manifestations of genetic syndrome. We excluded cases of patients, despite being diagnosed with the above-mentioned syndromes, without keratoacanthoma-like appearance lesions and cases in which the investigation, the diagnostic approach, and the treatment plan were not adequately reported.

## 4. Results

The following tables present the age/gender of the case reports, the lesion descriptions and locations, and the treatment followed in each case (Table 2, Table 3, Table 4 and Table 5).

We found 13 patients’ case reports that were diagnosed with MSSE or Ferguson–Smith syndrome. Females and men were found with a ratio to 9 to 4 with an age range from 27 to 75 years old. The most cases were diagnosed based on the clinical appearance of multiple squamous cancers in early age or family history of squamous carcinomas rather than genetic-based methods. Most cases presented the classic image of keratoacanthomas (dome-shaped lesion with keratin plug). It is observed that the use of the terms keratoacanthoma or multiple self-resolving epithelioma or well-differentiated squamous cell carcinoma seem to be confused or, in some studies, identified as the same, especially in the early ones, indicating the early lack of knowledge towards the specific type of lesions [10]. Some patients also seem to develop more aggressive types of keratoacanthoma such as keratoacanthoma marginarum or malignant carcinoma types in the form of invasive squamous carcinoma [16]. The question arises as to whether a lesion that develops in a patient with such a syndrome can be identified as malignant and excised or can be identified as a keratoacanthoma type and resolve with time. Some authors also preferred the surgical procedure option in the case of keratoacanthomas that were located in dangerous zones such as nasal nostrils [10]. Also, one study indicated the improvement of lesions with the simultaneous improvement of a systemic disease such as myelodysplastic syndrome, indicating a possible connection [14]. Radiotherapy in some cases provoked the appearance of more lesions, while it was effective in malignant cases [13]. Most cases showed different first treatment options such as isotretinoin [12,15]. However, the recent ones conclude that the use of acitretin in different doses with or without the application of a topical treatment such as imiquimod was the most efficacious way to deal with the lesions [11,12,16,17]. Also, in one study which described more aggressive subtypes of keratoacanthomas, cetuximab and cisplatin were considered as therapeutic options [18].

**Table 3:** Cases reported to have eruptive keratoacanthoma of Grzybowski.

Study	Diagnosis	Syndrome	Age/Gender	Lesion Description	Lesion Location	Treatment Plan
[19]	Clinical presentation	Eruptive keratoacanthoma of Grzybowski	80/F	Numerous follicular nodules and crateform tumors	KAs: the legs, arms, and trunk and limbs	Surgical removal of the largest skin lesions, oral acitretin (0.5 mg/kg/day)
[20]	Clinical presentation	Eruptive keratoacanthoma of Grzybowski	64/M	Umbilicated papules and small follicular papules	KAs: face, upper limbs, and trunk; the oral mucosa	Retinoids (no specific treatment reported)
[21]	Clinical presentation	Eruptive keratoacanthoma of Grzybowski	70/F	Numerous follicular papules and several erythematous nodules with keratotic centers	KAs: upper limbs, lower limbs, and trunk	Surgical removal of the largest skin lesions Initial treatment that failed: oral acitretin (0.5 mg/kg/day) Treatment that succeeded: subcutaneous methotrexate (MTX) 15 mg/week
[22]	Clinical presentation	Eruptive keratoacanthoma of Grzybowski	63/M	Hyperkeratotic erythematous nodules	KAs: ear lobe, cheeks, nose, arm, shoulder	Complete excision, sorafenib withdrawal
[23]	Clinical presentation	Eruptive keratoacanthoma of Grzybowski	51/M	Scaly and ulcerated papules and nodules	KAs: upper and lower extremities	Topical imiquimod 5% cream once daily on five continuous days of the week plus lapacho tea dressings
[24]	Clinical presentation	Eruptive keratoacanthoma of Grzybowski	83/F	Multiple skin-colored to erythematous to brownish papules, with a keratotic center	KAs: face	Acitretin up to 25 mg daily
[25]	Clinical presentation	Eruptive keratoacanthoma of Grzybowski	46/F	Keratotic, dome-shaped, follicular-based papules and scattered crateriform keratotic papules	KAs: trunk and extremities	Photodynamic therapy, oral acitretin up to 25 mg daily
[26]	Clinical presentation	Eruptive keratoacanthoma of Grzybowski	66/M	Large keratotic papules	KAs: face, neck, and chest	Acitretin up to 25 mg/d and doxepin
[27]	Clinical presentation	Eruptive keratoacanthoma of Grzybowski	47/F	Skin-colored follicular papules, papules with central umbilication, and larger lesions with a central crater	KAs: face, neck, and trunk	Initial failed treatment approach: acitretin (firstly) and methotrexate (secondly) Cyclophosphamide pulse therapy was then started at a dose of 1 g monthly
[27]	Clinical presentation	Eruptive keratoacanthoma of Grzybowski	59/F	Skin-colored follicular papules, papules with central umbilication, and larger lesions with a central crater	KAs: face, neck, and trunk	Initial failed treatment approach: acitretin (firstly) and methotrexate (secondly) Cyclophosphamide pulse therapy was then started at a dose of 1 g monthly
[27]	Clinical presentation	Eruptive keratoacanthoma of Grzybowski	68/M	Skin-colored follicular papules, papules with central umbilication, and larger lesions with a central crater	KAs: face, neck, and trunk	Initial failed treatment approach: acitretin Lost to follow up
[28]	Clinical presentation	Eruptive keratoacanthoma of Grzybowski	57/F	Multiple papules	KAs: upper trunk, arms and hands	Excision of large lesions Acitretin up to 25 mg daily; cyclophosphamide up to 200 mg daily
[29]	Clinical presentation	Eruptive keratoacanthoma of Grzybowski	71/F	Scattered, hyperkeratotic papules with central ulcers	KAs: right wrist and anterior aspects of the shins	Excision (Mohs’ micrographic surgery)
[7]	Clinical presentation	Eruptive keratoacanthoma of Grzybowski	58/M	Multiple pruritic papules and nodular and papular erythematous lesions	KAs: head, trunk, neck, and shoulder	Initial failed treatment approach: acitretin, 1 mg kg daily; methotrexate, 15 mg weekly; subcutaneous interferon a-2a and psoralen plus ultraviolet (UV) A therapy No successful treatment plan reported

KA: keratoacanthoma.

The clinical presentation of eruptive keratoacanthoma of Grzybowski is mainly based on the numerous lesions and the itchy character of those lesions compared to MSSE. Also, usually in the description of the patients’ lesions, the authors use the term papules aligned with other bigger lesions with crateform appearances. In many cases [19,20], keratoacanthoma lesions were found to be associated with HPV subtypes such as HPV39, which is rarely found in healthy skin, and HPV16. Meanwhile, in one case, the syndrome was triggered by the use of antineoplastic treatment—sorafenib [22]. As for the treatment plans followed, the most common treatment approach was, as in the case of MSSE, oral acitretin. Also, in the case of present comorbidities, a treatment option that simultaneously treats another disease (such as methotrexate in the case of rheumatoid arthritis) should be preferred [21].

**Table 4:** Cases reported to have Muir–Torre syndrome.

Study	Diagnosis	Syndrome	Age/Gender	Lesion Description	Lesion Location	Treatment Plan
[30]	Heterozygote mutation of the MSH2 gene	Muir–Torre syndrome	35/M	Large-growing nodule	Well-differentiated cSCC or KA: cheek	Excision and plastic reconstruction
[31]	Deletion in the MSH2 MMR gene	Muir–Torre syndrome	41/M	Keratotic tumor with central crust	KA: finger (thumbnail)	Excision and curettage
[32]	Clinical presentation (existence of multiple cancers)	Muir–Torre syndrome	53/M	Small nodule	cSCC: nasal nostril	Resection
[33]	Mutations in MSH6 and MLH1	Muir–Torre syndrome and Turcot syndrome	49/M	New dome-shaped nodule	cSCC: antecubital fossa	Pembrolizumab (200 mg)
[34]	Clinical presentations (medical history of occurrence of multiple cancers)	Muir–Torre syndrome	60/M	Dome-shaped lesions	KA: penis and face	Excision
[35]	Mutation and loss of hMSH2	Muir–Torre syndrome	56/F	Large, irregularly shaped crater filled with keratin	cSCC-KA: face	Resection

KA: keratoacanthoma, cSCC: cutaneous squamous cell carcinoma.

Most cases of Muir–Torre as described are not presented with multiple KA such as MSSE and the eruptive Grzybowski variant. However, due to the appearance of multiple skin nodules, some of them with sebaceous differentiation, multiple keratoaconthoma syndrome cannot be ruled out due to its histological differentiation. Muir–Torre, as indicated by the cases we found, is a syndrome associated, except from keratoacanthomas or cSCC carcinomas, with other types of cutaneous neoplasia such as liposarcomas or cSCC or sebaceous carcinomas and the presence of internal organ carcinoma (colon, urothelial, or endometrial carcinoma) [32,34]. Therefore, the investigation of internal malignancy is of great importance in such patients. Also, in many studies, the appearance of keratotic cancers can be the first sign of this syndrome. Many of the patients described above revealed family history with relevant clinical presentation of a family member. The majority of patients were males, and the main treatment option was excision of the lesion. Clinicians also suggested the treatment of such lesions with anti PD-1 analogs to target both keratinizing and other organs cancers [33].

**Table 5:** Cases reported to have incontinentia pigmenti.

Study	Diagnosis	Syndrome	Age/Gender	Lesion Description	Lesion Location	Treatment
[36]	Clinical presentation	Incontinentia pigmenti	54/F	KAs: discolored patches of skin	Nail bed and fingers	Ablation or excision of the underlying nail bed with a subsequent skin graft
[9]	Clinical presentation	Incontinentia pigmenti	17/F	KAs: multiple large hyperkeratotic tumors within Blaschkoid hyperpigmented patches	Leg	Intralesional methotrexate

KA: keratoacanthoma.

According to the above mentioned cases, keratoacanthomatous lesions in incontinentia pigmenti have been described in the late stages of the disease. The main lesions were painful subungual tumors. Those lesions were also noticed in other cases of incontinentia pigmenti [37] that are not included in our research, as it contained studies dated after 2000.

## 5. Discussion

Keratoacanthomas (KAs) are tumors originating from keratinocytes in the follicular infundibular/isthmic region and are commonly found in sun-exposed areas of the body. They follow a three-phase pattern of development: a proliferative phase (early), a stabilized phase (well developed), and a regressive phase (late), resembling the respective hair cycle. KAs can also occur in individuals with darker skin colors, typically appearing as classic dome-shaped lesions with a central crater, often displaying pigmentation more frequently. The debate among experts centers on whether keratoacanthoma should be considered a sub-type of squamous cell carcinoma (SCC). In clinical practice, the majority of cutaneous lesions exhibiting characteristics of KAs are biopsied rather than observed for regression. This is mainly due to uncertainty regarding their malignant potential [3]. However, these biopsies are often partial, making it difficult to assess the entire lesion. The primary distinctions between SCC and KA lie in their biological behavior, including differences in terms of chromosomal abnormalities and neovascularization. The ambiguity surrounding keratoacanthomas extends to keratoacanthoma syndromes such as Ferguson–Smith, with some authors considering multiple self-healing squamous epithelioma (MSSE) a distinct entity, further complicating the existing data. This term confusion becomes more pronounced in older case reports where the terms SCC, KA, or MSSE often appear interchangeably. Distinguishing KA-like SCC or KA from invasive SCCs is crucial for making appropriate clinical decisions and managing patients. Therefore, most cutaneous lesions displaying features of KAs are biopsied rather than observed for regression, primarily due to uncertainty about their malignant potential, and the use of immunohistochemistry stains has been suggested to help diagnosis [38]. As biological behavior cannot be definitively predicted based solely on histopathology, especially in cases of partial biopsies, the excision of large lesions, especially in high-risk areas, is recommended and is followed in most cases reported.

In most of the above-mentioned cases, the diagnosis was based on the clinical presentation of the patient, meaning the appearance of multiple KA-like lesions and the report of a family member with similar symptoms. MSSE stands as the predominant type among multiple KAs. Its initial description dates to 1934 within Scottish families. Nevertheless, isolated occurrences of this condition have been documented across different nations. Typically, multiple self-healing lesions manifest during childhood, adolescence, or early adulthood, and sporadic and familial forms have been documented [10]. The age of patients presented above ranged from 27 to 75; however. this is not the age of the disease onset but the age of the patients at the time they visited a doctor to seek medical advice. It is known that the syndrome affects both males and females equally; however, we found a male to female ratio of 4:9. According to data we found, the location of the KAs is not specific, ranging from the face to extremities. Also, in most cases, as presented in our case report, clinicians chose oral acitretin (up to 20 mg) and the excision lesions that were larger or susceptible to an invasive or another more aggressive SCC subtype. Those lesions can be separated from KAs in appearance, as they do not have the classic KA appearance and they are presented as scaly nodes or plaques or soft, ulcerated, or hemorrhagic lesions. Therefore, clinicians should observe every lesion of MSSE patients that fails to regress and differs from the others.

As in the cases we reported, the appearance of multiple papules along with the classic crateform lesions leads to the diagnosis of Grzybowski’s generalized eruptive keratoacanthoma [28]. On the contrary, the existence or previous medical history of internal malignancy and sebaceous lesions makes clinicians more suspicious for Muir–Torre syndrome [35].

Worth mentioning is the term eruptive squamous atypia (ESA), which is used to describe an idiopathic condition involving the occasional development of atypical yet well-differentiated keratinocytes (also referred to as eruptive keratoacanthoma). ESA should be suspected, instead of a genetic syndrome, by the existence of an irritating trigger that could cause KA presentation and is often misdiagnosed as cancer and treated with excisional surgery, which in turn can further trigger the Koebner phenomenon. Foxton et al. presented an 87-year-old woman with eruptive keratoacanthomas on the lower legs being triggered by recent imiquimod therapy. The patient was treated with oral acitretin, up to 40 mg [39]. The authors based the KA appearance on the local irritation of the skin, akin to a Köbner phenomenon, or by the activation of the NF-kB pathway with the subsequent upregulation of genes promoting tumorigenesis. The NF-kB pathway is emerging as being of central importance in promoting resistance to apoptotic death, inflammation, and therefore DNA damage and cancer. This suggests a trigger-based pathophysiology of the disease rather than genetic like the syndromes we discussed above. Therefore, it is important to distinguish these entities and to avoid local treatment or surgery that in the case of ESA could lead to the production of further KA-like lesions [40].

Acitretin was the medication used in our cases, as well as in the most cases of MSSE and eruptive KA of Grzybowski, and even in ESA, leading to satisfactory results concerning the appearance of KAs. Acitretin contributes to KA treatment by its anti-inflammatory and anti-proliferative effects, normalizing keratinocyte differentiation in the epithelium [41]. Oral acitretin can also reverse Wnt (Wingless-related integration site)-related KA proliferation as Wnt is a gene found to be activated in the growth phase and inactivated in the regression phase [42]. Usually, acitretin is used in combination with the excision of larger lesions, and in the case of failure, methotrexate and cyclophosphamide are utilized. Anti-PD-1 treatment was preferred in Muir–Torre syndrome with the aim to also treat internal malignancies [33]. Also, the two birds with one stone strategy was followed in cases of a co-existing systematic disease like rheumatoid arthritis in the case of methotrexate use [21]. Methotrexate, effective for the treatment of rapidly growing tumors by inhibiting DNA synthesis in actively dividing cells, serves as an alternative to surgical treatment for KAs. The intralesional use of methotrexate offers a less invasive and cost-effective approach with reduced morbidity in the case of well-differentiated KA-type lesions [43]. However, clinicians should be aware of the adverse effects of these drugs. For example, acitretin can be responsible for hepatotoxic reactions and the alteration of the lipid profile and symptoms such as blurred vision, headaches, and reduced night vision. As a retinoid, the overdose symptoms are similar to vitamin A toxicity and include headaches, nausea, vomiting, drowsiness, and vertigo [41]. In the case of large KAs that did not exhibit cutaneous manifestations of malignancy and for which their surgical treatment was not plausible, Ambur et al., in their review, outlined that data concerning appropriate alternative therapy for KAs are lacking [44].

## 6. Conclusions

The KA-like SCC or KA or MSSE terms need to be further separated in terms of clinical and histopathology assessment as well as biologic behavior so that clinicians are directed in their diagnosis and in the treatment approach of a patient with one or multiple KA-like lesions. The possibly of ESA should be kept in mind when clinicians use topical treatment, while a lesion that does not regress and differs in the case of MSSE should be treated with rising suspicion of a more aggressive malignancy. The presence of sebaceus lesions or multiple papules along with KA-like lesions and internal malignancies reports direct to Muir–Torre diagnosis. As in our case, in most cases reported in MSSE and eruptive keratoacanthoma of Grzybowski variants, oral acitretin was used, and excision was used in cases of larger and more aggressive lesions (invasive SCCs). Low doses of acitretin constitute an important, safe, and effective choice in the treatment of multiple therapy-resistant keratoacanthomas. The combination of acitretin with topical 5-fluorouracil is a safe treatment option which allows the use of minimal doses of acitretin and the faster achievement of the therapeutic goal.

## Figures and Tables

**Table 1:** The differential aspects (clinic, histopathologic, and evolutive) of KA lesion multiple KA-like syndromes.

KA-like Syndromes	Clinical	Histopathological	Evolutive
Ferguson–Smith syndrome (MSSE)	Multiple large KA-like lesions in sun-exposed areas	KA histopathology features with no marked “shouldering” and the leucocyte abscesses are absent	Undergo spontaneous regression, resulting in the formation of atrophic scars
Eruptive keratoacanthoma of Grzybowski	Numerous KA-like itchy papules with hardened centers	KA histopathology features	Persistence if not treated
Multiple familial keratoacanthoma of Witten and Zak	Combined large and small KA-like lesions (with overlapping features of the Ferguson–Smith and Grzybowski subtypes)	KA histopathology features as in MSSE or eruptive keratoacanthoma of Grzybowski	Shared evolutionary characteristics between the Ferguson–Smith and Grzybowski KA subtypes
Incontinentia pigmenti	Multiple large hyperkeratotic KA-like lesions within Blaschkoid hyperpigmented patches	KA histopathology features	Persistence if not treated
Muir–Torre syndrome	KA lesions often accompanied by sebaceous epitheliomas or carcinomas	KA-like histopathology features along with sebaceous characteristics	Persistence if not treated

KA: keratoacanthoma.

**Table 2:** Cases reported to have MSSE or Ferguson–Smith disease.

Study	Diagnosis	Syndrome	Age/Gender	Lesion Description	Lesion Location	Treatment Plans Described
[10]	Family history	MSSE	51/F	Dome-shaped, yellowish-red nodule with a central hyperkeratotic plug	KA-like lesions: face/arms (preferably)/hands/nose Well-differentiated SCC: upper lip/close to nose	Surgical excision or non-intervention led to regression X-ray treatment
[10]	Family history	MSSE	75/M	NA	Not specific type (invasive or KA like tumor): forehead	X-ray treatment
[10]	Family history/early stage of skin cancer appearance	MSSE	43/F	Dome-shaped, yellowish-red, scaly, with centrally crusted lesions	KA-like lesions: upper lip, nose and chin Well differentiated SCC: upper lip/close to nose Invasive SCC: right cheek	Non-intervention or imiquimod led to regression or excision. Excision and plastic correction Excision
[10]	Family history	MSSE	34/F	A flat, keratotic tumor	KA-like lesions: cheek	Excision
[10]	Family history	MSSE	41/M	NA	KA-like lesions: forehead	Curettage
[10]	Family history	MSSE	50/F	Dome-shaped tumor	KA-like lesions: cheek	Non-intervention led to regression
[11]	Family history	MSSE	49/F	NA	SCC: basal vestibule KA-like lesions and SCC: midface	Radiotherapy led to regression Interferon-a failed to lead to regression Acitretin (up to 60 mg daily for 12 weeks) led to improvement
[12]	TGFBR1 mutation	MSSE	37/F	Ulcerated lesions or nodular keratinized firm nodules	Well-differentiated, moderately invasive, and keratoacanthoma-like lesions: upper lip and cheeks	Isotretinoin and excision choices failed patient consent Acitretin (10 mg daily) led to-regression
[13]	Clinical image	MSSE	60/F	A firm, skin-colored, centrally keratin-filled dome-shaped nodule	Radiotherapy-induced KA-like lesions: hand.	Minimal surgical intervention combined with intralesional injections of triamcinolone acetate led to improvement
[14]	Clinical image	MSSE	47/F	Three flesh-pink, sharply circumscribed, crateriform nodules with keratotic cores	KA-like lesions: the right eyebrow and the right auricula	5% imiquimod cream and better control of myelodysplastic syndrome—a systemic disease)
[15]	Clinical presentation (non-familial)	MSSE	27/F	Multiple keratotic lesions	KA-like lesions: holes and soles	No treatment led to regression Isotretinoin and topical application of tazarotene—failed and switched to acitretin, up to 35 mg, which had better results
[16]	Not reported	MSSE and keratoacanthoma centrifugum marginatum	46/M	Multiple pruritic, keratotic popular, and nodular eruptions An annular, hyperkeratotic verruciform plaque	KA-like lesions: shins, forearms, and buttocks KA centrifugum marginatum: the dorsum of the right foot	Acitretin (0.7 mg/kg/day) led to regression
[17]	Clinical presentation	MSSE	41/M	Multiple dome-shaped, cutaneous nodules with disfiguring scars	KAs: trunk and limbs	Oral acitretin at 20 mg/day led to regression

MSSE: multiple self-healing squamous epithelioma, NA: not applicable, KA: keratoacanthoma.

## Data Availability

The data described in this study are available upon request from the corresponding author.
